# Impact of the COVID-19 Pandemic on Head and Neck Cancer Management: The Experience of the Maxillo-Facial Surgery Department of a French Regional Referral Center in a High-Incidence Area

**DOI:** 10.3390/jcm13082439

**Published:** 2024-04-22

**Authors:** Emilien Colin, Agnès Paasche, Alban Destrez, Bernard Devauchelle, Jérémie Bettoni, Julien Bouquet, Stéphanie Dakpé, Sylvie Testelin

**Affiliations:** 1 Maxillofacial Surgery Department, Amiens University Hospital, Rond-Point du Pr Christian Cabrol, 80054 Amiens, France; paasche.agnes@chu-amiens.fr (A.P.); destrez.alban@chu-amiens.fr (A.D.); devauchelle.bernard@chu-amiens.fr (B.D.); bettoni.jeremie@chu-amiens.fr (J.B.); bouquet.julien@chu-amiens.fr (J.B.); dakpe.stephanie@chu-amiens.fr (S.D.); testelin.sylvie@chu-amiens.fr (S.T.); 2 UR 7516 CHIMERE, University of Picardie Jules Verne, Chemin du Thil, CS 52501, 80025 Amiens, France; 3 Institut Faire Faces, Rond-Point du Pr Christian Cabrol, 80054 Amiens, France

**Keywords:** clinical outcomes, COVID-19, head and neck cancer, lockdown period, maxillofacial surgery, SARS-CoV-2

## Abstract

**Background:** Cancer patients are at a high risk of complications in cases of infection, and head and neck cancers (HNC) are no exception. Since late 2019, SARS-CoV-2 has caused a global health crisis, with high rates and severe forms of the disease in cancer patients. Hospitalization, surgery and radiotherapy were rapidly described as increasing the risk of infection. Since March 2020, the Amiens University Hospital (France) has been taking care of COVID-19 patients while its maxillofacial surgery department managed HNC patients without interruption, even during lockdown periods. However, many questions concerning the impact on patient care were still pending. The aim of this study is to describe HNC management in our center during the first epidemic peak and to evaluate the impact of containment measures on patient treatment. **Methods**: We retrospectively included 44 HNC patients treated in our department between 1 March and 31 August 2020. Two groups were defined according to the period of care: lockdown (March to May) and lighter restrictions (June to August). **Results**: The results show typical epidemiological characteristics, maintained management times and non-downgraded procedures. **Conclusions**: Thus, during the first epidemic peak, continuity of care and patients’ safety could be ensured thanks to adequate means, adapted procedures and an experienced surgical team.

## 1. Introduction

Since its emergence in late December 2019, SARS-CoV-2 has rapidly spread, causing a global health crisis [1]. The disease, caused by this highly contagious respiratory virus, is called COVID-19. By end of June 2023, more than 700 million cases were reported worldwide, including over 275 million in Europe, causing more than 6.9 million deaths worldwide and over 2.2 million in Europe (source: https://www.ecdc.europa.eu, accessed on 31 January 2024), as the virus can cause acute respiratory distress syndrome (ARDS) and potentially fatal multiple organ failure [2,3]. Cancer patients are generally susceptible to infections and especially to respiratory viruses [4], and SARS-CoV-2 appears to be no exception. Indeed, early Chinese studies reported a higher rate of SARS-CoV-2 infection in people with current cancer or cancer history than in the general population, with more severe forms and an increased risk if surgery or chemotherapy were performed in the month prior to infection. These studies concluded that hospitalization and regular consultations are potential risk factors for COVID-19 infection in cancer patients [5,6].

Head and neck cancers (HNC) are a heterogeneous group of tumors whose location often involves anatomical structures of the upper aero-digestive tract, such as the oral cavity, larynx and oropharynx [7]. These cancers represent a global public health issue [8], particularly in Europe, with 128,600 new cases of lip, oral cavity or oropharynx cancers in 2020 [9]. Especially, France presents some of the highest incidence and mortality rates, with the north of the country showing a significant over-incidence of oral cavity cancers (OCC), the Hauts-de-France region being the most affected [10]. Squamous cell carcinomas (SCC) are the most common type of OCC, with approximately 90% of cases [7]. The majority of them concern men over 50 years old [11], but with a global incidence showing an increasing trend in younger people and in women [12]. The main risk factors for SCC of the upper aero-digestive tract are alcohol and tobacco consumption and exposure to high-risk human papillomavirus (mostly HPV-16 and HPV-18) [13,14]. Despite their frequency, these cancers are often diagnosed at an advanced stage [15], even for locations accessible to visual or tactile examination [16]. Currently—with the exception of HPV-related cancers—they still present a poor prognosis, with a 5-year survival of approximately 50% [17] and a high risk of local recurrence and/or distant metastasis within a few years after diagnosis [18], depending on the location and TNM stage. Upper aero-digestive tract SCC is the most frequent HNC, and the most common first-line treatment for HNC is therefore surgery [7], with the necessity to perform microsurgical reconstruction using free flaps in many cases, including all the risks it entails under current conditions [19,20,21].

At the time of the first epidemic peak in Europe, only a few studies measured the impact of COVID-19 on the management of HNC, even though patients were particularly exposed due to the location of their tumors and the procedures for their care. The complexity of defining an optimal therapeutic strategy during the pandemic was emphasized, and authors recommended an individual global benefit/risk ratio assessment in order to protect patients while avoiding any loss of chance with regard to the treatment of their cancer [22]. Another question concerned the development of new organizational strategies to guarantee both patient care and staff safety [23]. Since then, numerous studies dealing with COVID-19 and its consequences on health systems organization and patients’ management have been published in all of the international medical journals, whatever the specialty [24]. Head and neck cancer is no exception, and many studies have been published, whether they attempt to assess the impact of coronavirus on patient outcomes [25,26] or on surgical practice [27,28] or whether they aim to provide recommendations both for patient management [29,30,31] and for the protection of healthcare workers particularly exposed to the risk of contamination [32,33].

The Amiens University Hospital is a regional tertiary referral center—namely, in the field of oncology—and its maxillofacial surgery department has been internationally renowned since the first partial face transplant in 2005 [34,35]. The department attracts numerous head and neck cancer patients, predominantly in advanced stages, and especially patients with recurrent disease, which may require large surgical resections imposing free flap reconstructions and complementary treatments, depending on the disease and the patient’s history. Since the end of February 2020 and the beginning of the pandemic in France, the Amiens University Hospital has been taking care of COVID-19 patients while maintaining its oncology activity, also during the different lockdown periods, the first and most restrictive of which lasted from March to May 2020. While most non-cancer surgeries were massively postponed and medical consultations were cancelled or arranged online, the maxillofacial surgery department continued to manage cancer patients, especially since operating theatres remained accessible only for cancer surgery. However, many questions were still pending at this time, including the risk of SARS-CoV-2 infection and the severity of the COVID-19 disease in this high-risk population, but also regarding the impact of the crisis on waiting times in patients’ care and on the treatment options at this time. Such considerations were particularly legitimate in our department, the Hauts-de-France region having the highest national incidence of oral cavity cancers and being among the French territories most affected by COVID-19 at this time (source: https://www.santepubliquefrance.fr, accessed on 31 January 2024).

The aim of this study is to describe HNC management in our center during the first epidemic peak and to evaluate the impact (i) of the pandemic and (ii) of containment measures, in effect at the time, on patient treatment.

## 2. Materials and Methods

### 2.1. Study Design

This is a retrospective study including patients, 18 years of age and older, diagnosed with upper aero-digestive tract cancer and treated in the maxillofacial surgery department of the Amiens University Hospital between 1 March and 31 August 2020. Patients under 18 years of age, patients with head and neck cancer of another location and patients whose treatment decision had been made before the inclusion period were excluded. Three senior and two junior surgeons led the multidisciplinary team meetings (MTM) and performed the surgeries.

The selected patients were considered in totality over the 6-month inclusion period (from March to August 2020) and by group according to the period of care: from March to May 2020 (group 1) and from June to August 2020 (group 2). Indeed, group 1 corresponds to the beginning of the epidemic in France and includes in particular the first full lockdown period (17 March to 10 May 2020), and group 2 corresponds to the reopening of the country, while maintaining lighter restrictions.

This study has been approved by the local ethical committee of the Amiens University Hospital on 4 January 2021 (reference PI2021_843_0003) and was registered on ClinicalTrials.gov (NCT04704466). In accordance with the French Public Health Code, all patients were informed at the time of admission that their regular medical data may be used for research purposes, and none of them had expressed opposition to the processing of their data. This study was registered with the CNIL (French personal data protection agency) under the reference methodology MR004.

### 2.2. Collected Data

Data were extracted from the electronical medical files and included the patient’s date of birth, sex, history of previous cancer, date of diagnosis for current cancer, tumor topography and histological type, immuno-histochemical status with respect to the p16 protein, clinical stage of the tumor at diagnosis (TNM stage according to the 8th edition of the UICC TNM Classification), date of the multidisciplinary team meeting (MTM), treatment decision, actually performed treatment, reasons for non-performing or for modifying treatment, first treatment start date, type of surgical procedure, complications, reconstruction data with flap type and outcomes, hospitalization duration, SARS-CoV-2 testing and results, follow-up data with survival at 6 and 12 months and cancer status at 12 months.

### 2.3. Care Waiting Times

The Diagnosis to Multidisciplinary Team Meeting Interval (DMI), Multidisciplinary Team Meeting to first-Treatment Interval (MTI) and Diagnosis to first-Treatment Interval (DTI) were calculated in days. The date of diagnosis was considered the first histological sampling confirming the cancerous nature of the disease. In the absence of tumor biopsy prior to treatment, the date of diagnosis was considered to be the date of first admission to the hospital for a medical imaging examination or a consultation resulting in the patient being referred to oncology, or if failing that, the first MTM date.

### 2.4. Data Analysis

For descriptive analysis, quantitative data are described using the mean ± standard deviation and the median (minimum–maximum). Qualitative data are described by frequency as a percentage of cases. For comparative analysis, quantitative data are compared using a Student’s *t* test for independent samples if data follow a normal distribution or a Mann–Whitney test if not. Qualitative data are compared using Fisher’s exact test due to the small size of each group. Analysis was performed using Prism 9 software (GraphPad Software Inc., San Diego, CA, USA).

## 3. Results

### 3.1. Patients’ Data

A total number of 44 patients (29 men and 15 women) were included over the 6-month period, with a median age of 66.7 years old [41.2 to 92.9]. Of these, 79.6% were current or former smokers, 63.6% were current or former regular alcohol users and 61.4% had the two associated risk factors. Patients had a history of cancer in 52.3% of cases, and 50.0% had a history of upper aero-digestive tract cancer. All patients’ data are reported and detailed, according to the period of care, in Table 1.

### 3.2. Cancer Data

The most frequent cancer topography was tongue, with 29.6% of cases, followed by mandible and/or floor of the mouth (22.7%), maxilla and/or palate (15.9%), parotid gland (11.4%), oropharynx (6.8%), inner side of cheek (6.8%) and other (6.8%). The tumor histology was squamous cell carcinoma in 93.2% of cases, muco-epidermoid carcinoma (4.6%) and verrucous carcinoma (2.3%). Immuno-histochemical staining for the p16 protein was performed in 56.8% of cases, of which 8.0% showed overexpression. A total of 30.0% were localized tumors (T1 or T2) and 70.0% were extended tumors (T3 or T4) according to the 8th edition of the UICC TNM Classification, corresponding to 27.5% of early-stage cancers (I–II) and 72.5% of advanced stage cancers (III–IV), the statistical analysis showing no significant differences in the tumor classification or cancer stage between the two groups. All cancer data are reported and detailed, according to the period of care, in Table 2.

### 3.3. Patients’ Management

In total, 90.9% of patients were able to benefit from a multidisciplinary team meeting (MTM), including 86.4% of patients in group 1 and 95.5% of patients in group 2, with no significant difference (*p* = 0.6). Regarding the treatment decision, 72.7% of patients were treated surgically, this proportion being the same in both groups. Radiotherapy, alone or in association with another treatment, was decided for 65.9% of patients (72.7% in group 1 and 59.1% in group 2, *p* = 0.53), and chemotherapy was decided in 38.6% of cases (45.5% in group 1 versus 31.8% in group 2, *p* = 0.54). The complete treatment regimens included exclusive surgery in 29.6% of cases, surgery with complementary radiotherapy in 29.6% of cases, chemo-radiotherapy in 20.5% of cases, surgery with complementary chemo-radiotherapy in 13.6% of cases, chemotherapy alone in 4.6% of cases and radiotherapy alone in 2.3% of cases. Of these, 100.0% of planned surgeries were performed, compared to 75.9% of radiotherapies (87.5% in group 1 versus 61.5%, *p* = 0.19) and 76.5% of chemotherapies (90.0% in group 1 versus 57.1% in group 2, *p* = 0.25). Treatments not performed, concerning only radiotherapy and chemotherapy, were in the majority due to cancer progression (55.6% of cases), with the deterioration of the general condition in patients before the start of treatment, making it impossible to perform. The other reasons were a reassessment of patients’ conditions in the oncology department, leading to the decision not to carry out chemotherapy, especially because of their advanced age (two patients, 22.2% of cases) and the patient’s refusal of treatment (two patients, 22.2% of cases). All patients’ management modalities are reported and detailed, according to the period of care, in Table 3.

### 3.4. Care Delays

**Figure 1:**
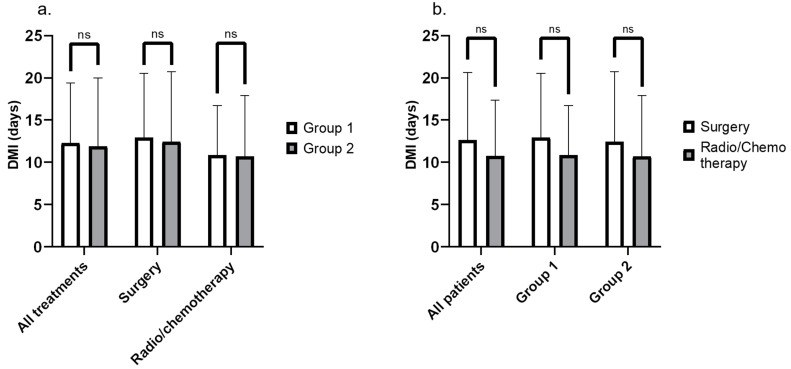
Diagnosis to the multidisciplinary team meeting (MTM) interval (DMI) according to the group (**a**) and according to the first treatment modality (**b**).

Considering all patients and all treatments, the median DMI was 10 days [1 to 34], the median MTI was 19 days [4 to 68] and the median DTI was 31 days [5 to 79]. DMI were compared between the two groups for all treatments, for surgery, and for other treatment modalities (Figure 1a). The DMI were also compared between treatment modalities for all patients and for each of the two groups (Figure 1b). Statistical analysis showed no difference in DMI, neither according to the period of care nor to the first treatment modality.

**Figure 2:**
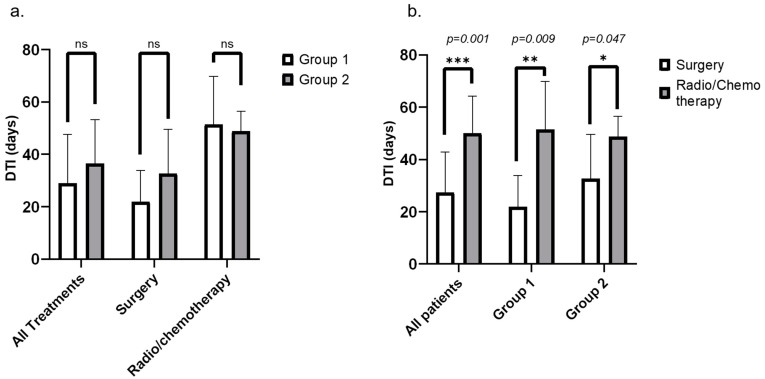
Diagnosis to treatment interval (DTI) (in days) according to the group (**a**) and according to the first treatment modality (**b**) (* significant, ** highly significant, *** very highly significant).

On the other hand, diagnosis to treatment intervals were compared between the two groups for all treatments, for surgery, and for other treatment modalities (Figure 2a), and the results again show no significant difference between the groups, whatever the treatment modality.

Finally, DTI were also compared between treatment modalities for all patients and for each of the two groups (Figure 2b). Statistical analysis showed significant differences in DTI for all patients (22.5 days [5–75] versus 46.5 days [26–79], *p* = 0.001) and both for group 1 (20.5 days [5 to 44] versus 47 days [26 to 79], *p* = 0.009) and for group 2 (31.5 days [12 to 75] versus 46 days [42 to 63], *p* = 0.047), care delays being inferior for surgery compared to other treatments modalities. All management delays (DMI, MTI and DTI) are reported and detailed, according to the period of care, in Table 4.

### 3.5. Surgical Management

All of the planned surgeries were performed, all of them under general anesthesia. The surgical procedures were glossectomy and pelvi-mandibulectomy (18.8% of cases each), maxillectomy (15.6%), pelvi-glosso-mandibulectomy (12.5%), parotidectomy (9.4%) and other (25.0%). Lymph node dissection was performed in 56.3% of cases, and 75.0% of patients underwent flap reconstruction, 87.5% of which were immediate reconstructions and 12.5% were secondary reconstructions. In two cases (8.3%), a double flap reconstruction was performed. Statistical analysis shows no difference in the number of reconstructions according to the period of care (68.8% for group 1 versus 81.3% for group 2, *p* = 0.685). Free flaps were used in 76.9% of cases, and local flaps were used in 23.1% of cases. Flap types were mostly radial forearm free flaps (38.5% of cases), fibula free flaps (23.1%), latissimus dorsi (19.2%, corresponding to five flaps, including one free flap and four pedicled flaps) and scapula free flaps (7.7%), with a total success rate of 88.5%. The median hospitalization duration was 12 days [2 to 79], with complications in 28.1% of cases, including surgical re-intervention in 12.5% of cases (flap failure in 9.4% and hemostasis verification in 3.1% of cases) and infection in 15.6% of cases (flap or surgical site infection in 6.3% and pulmonary infection in 9.4%). Of these, one death occurred in hospitalization (3.1%) due to pulmonary sepsis, cardiogenic shock and multivisceral failure, unrelated to SARS-CoV-2 infection, the patient having received several PCR tests during hospitalization. All surgical management data are reported and detailed, according to the period of care, in Table 5.

### 3.6. SARS-CoV-2

Within the total population, 45.5% of patients were screened for SARS-CoV-2 infection, all by PCR (100.0%), and none of them were positive (0.0%). In addition, the data show that group 2 patients were more likely to be screened than group 1 patients (63.6% versus 27.3%, *p* = 0.033). SARS-CoV-2 testing and infection data are reported and detailed, according to the period of care, in Table 6.

### 3.7. Patients’ Follow-Up

**Figure 3:**
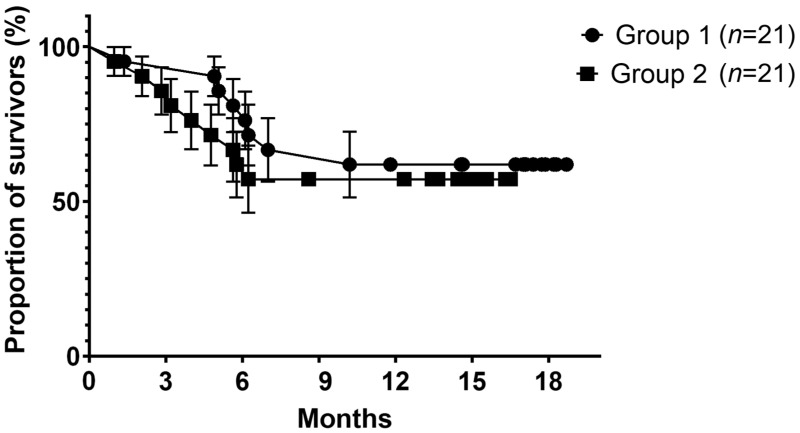
Proportion of survivors (%) for each group over time (months).

Within the total population, 38.1% of patients were in remission 12 months after treatment, and 61.9% either relapsed or progressed. Statistical analyses showed no difference in cancer status or survival—at 6 or 12 months—depending on the period of care. The Kaplan–Meier survival analysis is presented for the two groups in Figure 3. All patients’ follow-up data are reported and detailed, according to the period of care, in Table 7.

## 4. Discussion

The management of patients with HNC is always complex and was all the more challenging during the pandemic, causing stress regarding hospital capacity and imposing constant reorganization of the healthcare system. This retrospective study reports on patients with upper aero-digestive tract cancer during the early months of the pandemic: the initial period of full lockdown and the subsequent period of lighter restrictions. Our maxillofacial surgery department is located in a Regional University Hospital and is a nationally renowned tertiary referral center for head and neck pathologies [34,35]. With that being said, we routinely take care of patients with advanced head and neck cancer that require large surgical resections and complex microsurgical reconstruction. The retrospective analysis of our department activity during the first 6 months of the pandemic shows epidemiological characteristics that are usual in our area, with a continuity of care despite the situation.

Being a man, over the age of 50, with alcohol and tobacco consumption (Table 1) are common HNC patients’ characteristics in the north-west of France, as well as the trend towards recurrence [10,11]. The same applies to the most frequent locations (tongue, floor of the mouth), the histology (squamous cell carcinoma in a large majority of cases) and the usually advanced tumor stages (III–IV) at diagnosis [11,16]. Furthermore, all but one case in each group did not show an overexpression of the p16 protein, which is also in line with the usual characteristics of these cancers in our region. When comparing the data between groups (Table 2), the tumor T stage and the cancer stage did not differ significantly, suggesting that there was no more admission for the advanced stages after the lockdown than before. However, it must be considered that as a tertiary referral center, most of our cancer activity usually involves advanced cancer stages. The French recommendations for HNC management continued to be followed, with 90.91% of all patients having attended a multidisciplinary team meeting [36]. The fact that slightly fewer patients in group 1 benefited from an MTM than in group 2 (19 versus 21) is due to the fact that the MTM takes place once a week; therefore, during the pandemic, some surgical decisions may have been anticipated in order to save time and to take advantage of available operating rooms. It was not because MTM could not be organized due to COVID-19.

Overall, patients’ management data for the first 3 months are comparable with the following, suggesting that the full lockdown did not impact treatment decisions and care in our department during the first 3 months of the COVID-19 crisis and over the next 3 months (Table 3). Although the data did seem to show a decrease in radiotherapy and chemotherapy decisions in group 2, in favor of more exclusive surgeries, statistical analyses show no significant difference. Similarly, there seems to be a trend towards an increase in the number of non-surgical treatments not performed in group 2, but again, this difference is not significant. Perhaps this is due to the small sample size. Management delays for non-surgical treatments (DTI 50.1 ± 14.2 days, median 46.5) are in line with a previous study by Guizard et al. (2016), which described a median time of 54.5 days for radiotherapy treatment especially [37]. These delays do not seem to have been impacted by the full lockdown period in our center, with a median DTI of 47 days versus 46 for groups 1 and 2, respectively (Table 4). These waiting times, however, remain an issue in head and neck cancers’ management, since treatment delays have an impact on disease prognosis.

One result that seemed to emerge from our data was the delay to surgical management during lockdown, which tended to be shorter than during the following 3 months, with 21.94 ± 11.95 days versus 32.63 ± 16.95 days, respectively. This could easily be explained by the fact that, during the first months of the pandemic, and particularly during the full lockdown period, all elective surgeries were postponed apart from oncology. These unprecedented conditions explain this trend, but statistical analysis showed no significant difference (*p* = 0.083), perhaps because of the small size of the study. Other than that, for all patients and all treatments, the median DMI and DTI of, respectively, 10 and 31 days appear to be consistent with the Guizard et al. (2016) study on delays for HNC management in the north-west of France, which described median DMI and DTI values of 14 and 35 days, respectively [37]. However, our results seem to be in contradiction with a 2021 study from COVIDSurg Collaborative (in which our center participated), conducted in 466 hospitals of 61 countries with 15 cancer types. This study reports that one in seven patients in regions with a full lockdown did not undergo planned surgery or experienced longer preoperative delays. This potentially led to long-term reductions in survival [38]. It is important to keep in mind that these data concern different specialties, with head and neck cancers accounting for only 17.6% of cases. Thus, they are not necessarily representative of our specialty. In addition, a study by Tevetoğlu et al. (2021) reports more admissions for advanced-stage HNC, an increased use of complex reconstructions and a longer time to surgical treatment during the first 6 months of the pandemic in comparison to the same period the previous year [39], in contrast to the data of our center once again.

Treatment decisions taken in MTM were not modified in relation to our guidelines, and all decided surgeries were performed, including free flap reconstructions when necessary, in the same way as usual (Table 5). The hospitalization duration is comparable between the two groups and corresponds to the mean hospitalization duration in our department for these indications. The average success rate for flaps exceeds 88% in the total population, with two flap failures in group 1 and one flap failure in group 2. However, it is important to bear in mind that we are a referral center for HNC, and our patients may be complex cases with prior surgical or radiotherapy treatment (70% of them being with advanced cancer and 50% being in recurrence in this study). Considering that we used free flaps in nearly 77% of cases, our results suggest that the use of free flaps is possible even in periods of intense hospital stress. However, several studies dealing with head and neck reconstruction during the pandemic have been published, some recommending the use of pedicled flaps, which are less difficult to monitor [40,41]. Indeed, Rauso et al. (2021) emphasized that free flaps represent extremely specialized procedures that required resources that were not affordable during this period. In contrast, other authors consider that the gold standard should not be given up despite the pandemic conditions and that adapted protocols and the rigorous selection of patients should avoid downgrading management procedures [42,43]. This is in accordance with our experience. In the same way, a COVIDSurg Collaborative study (2020) dedicated to HNC surgery (in which our center participated as well) found evidence of surgical de-escalation in HNC management and reconstruction. However, it concluded that HNC surgery in the COVID-19 era appears to be safe even for prolonged and complex interventions, which is consistent with our results [44].

Concerning the screening of patients for SARS-CoV-2, our data reflect the absence of systematic testing at the beginning of the pandemic (with 27.27% of patients tested in group 1 versus 63.64% in group 2) and screening having become systematic only after a few months (Table 6). These results reflect variable recommendations over the study period, with the instruction to screen on a case-by-case basis during the summer of 2020. Indeed, PCR screening for all patients only became mandatory in France during the “second COVID wave” in the autumn, which is not covered by this study. None of our tested patients were positive, and it can be assumed that if some untested patients carried the virus, the health safety procedures applied at the time were effective.

In the end, the patients’ survival at 6 and 12 months shows no significant difference between the groups (69.05% and 59.52%, respectively). The recurrence rate remains consistent with what we usually observe in practice (Table 7), reminding us of the aggressive nature of oral SCC and of the poor prognosis for patients who are too often referred at advanced stages.

Of course, this study is a photograph illustrating the management of HNC in our department at a given time and on a small number of patients, which may raise questions about the comparability of the two groups (“lockdown” and “lighter restrictions”), each with about 20 patients. These results may therefore not be applicable to the other centers, even if a large COVIDSurg Collaborative study (2020) goes in the same direction on some points [44]. In order to increase the size of the study population, it might have been necessary to conduct a multicenter study in collaboration with other centers in our region. However, surgical practices and organization of care vary from one center to another, and this could have generated new biases. The heterogeneity of publications regarding HNC surgical management probably underlines the team-dependent (or operator-dependent) aspect of surgical practice, even more so during times of crisis and despite the various guidelines. If clinical outcomes depend on the surgeons’ experience and practice, they also depend (i) on the established procedures and (ii) on the means provided by institutions. It is also essential to highlight that not all countries or territories were impacted in the same way and at the same time by the pandemic and that governments’ decisions may also have differed. This could have influenced the way HNC patients were managed according to their area. Moreover, it could have been useful to collect data over the same period in 2019, in the manner employed by Tevetoğlu et al. [39], in order to provide a control group to the total population of our study. Finally, with the COVID-19 pandemic still ongoing several years later, another study comparing our current activity versus 2020/2021 could be interesting in assessing our institution’s long-term resilience to the health crisis.

## 5. Conclusions

With this retrospective monocentric study, we provide an overview of the management of patients with HNC during the first six months of the COVID-19 pandemic in a world-renowned maxillo-facial department within a regional tertiary referral center, taking care of COVID-19 patients while maintaining its cancer activity. Our results show that the usual waiting times for patient care were maintained and that our procedures have not been downgraded. This suggests that, thanks to the adequate means, adapted procedures and an experienced surgical team, it is possible to continue to treat HNC patients safely and according to the guidelines, even in times of pandemic crises.

## Figures and Tables

**Table 1:** Patients’ demographics, risk factors and cancer history for the entire study population and for each group.

	All Patients (*n =* 44) 1 March–31 August 2020	Group 1 (*n =* 22) 1 March–31 May 2020	Group 2 (*n =* 22) 1 June–31 August 2020
**Age**			
Mean ± SD (years)	67.6 ± 12.7	68.0 ± 12.3	67.2 ± 13.1
Median [range] (years)	66.7 [41.2–92.9]	66.1 [43.1–92.9]	68.6 [41.2–86.9]
**Genre**			
Men, *n* (%)	29 (65.9)	12 (54.6)	17 (77.3)
Women, *n* (%)	15 (34.1)	10 (45.5)	5 (22.7)
**Risk factors**			
Tobacco, *n* (%)	35 (79.6)	20 (90.9)	15 (68.2)
Alcohol, *n* (%)	28 (63.6)	17 (77.3)	11 (50.0)
Both, *n* (%)	27 (61.4)	17 (77.3)	10 (45.5)
**Previous cancer history**			
All types, *n* (%)	23 (52.3)	13 (59.1)	10 (45.5)
Head and Neck, *n* (%)	22 (50.0)	13 (59.1)	9 (40.9)

**Table 2:** Cancer location, histology, p16 status and tumor classification for the entire study population and for each group.

	All Patients (*n =* 44) 1 March–31 August 2020	Group 1 (*n =* 22) 1 March–31 May 2020	Group 2 (*n =* 22) 1 June–31 August 2020
**Location**			
Tongue, *n* (%)	13 (29.6)	2 (9.1)	11 (50.0)
Mouth floor/mandible, *n* (%)	10 (22.7)	7 (31.8)	3 (13.6)
Maxilla/palate, *n* (%)	7 (15.9)	6 (27.3)	1 (4.6)
Parotid gland, *n* (%)	5 (11.4)	3 (13.6)	2 (9.1)
Oropharynx, *n* (%)	3 (6.8)	0 (0.0)	3 (13.6)
Inner side of cheek	3 (6.8)	1 (4.6)	2 (9.1)
Other, *n* (%)	3 (6.8)	3 (13.6)	0 (0.0)
-Lip	1 (2.3)	1 (4.6)	0 (0.0)
-Nasal cavity	1 (2.3)	1 (4.6)	0 (0.0)
-Sinus	1 (2.3)	1 (4.6)	0 (0.0)
**Histology**			
Squamous cell carcinoma, *n* (%)	41 (93.2)	20 (90.9)	21 (95.5)
Muco-epidermoid, *n* (%)	2 (4.6)	2 (9.1)	0 (0.0)
Verrucous carcinoma, *n* (%)	1 (2.3)	0 (0.0)	1 (4.6)
**p16 protein**			
Tested, *n* (%)	25 (56.8)	11 (50.0)	14 (63.6)
-Positive, *n* (%)	2 (8.0)	1 (9.1)	1 (7.1)
**Tumor classification**	***n* = 40**	***n* = 20**	***n* = 20**
T1–T2, *n* (%)	12 (30.0)	7 (35.0)	5 (25.0)
T3–T4, *n* (%)	28 (70.0)	13 (65.0)	15 (75.0)

**Table 3:** Therapeutic strategies for the entire study population and for each group.

	All Patients (*n =* 44) 1 March–31 August 2020	Group 1 (*n =* 22) 1 March–31 May 2020	Group 2 (*n =* 22) 1 June–31 August 2020
**Multidisciplinary team meeting, *n* (%)**	40 (90.9)	19 (86.4)	21 (95.5)
**Proposed treatment modality**			
Surgery, *n* (%)	32 (72.7)	16 (72.7)	16 (72.7)
Radiotherapy, *n* (%)	29 (65.9)	16 (72.7)	13 (59.1)
Systemic therapy, *n* (%)	17 (38.6)	10 (45.5)	7 (31.8)
**Recommended treatment plan**			
Surgery alone, *n* (%)	13 (29.6)	4 (18.2)	9 (40.9)
Surgery + radiotherapy, *n* (%)	13 (29.6)	8 (36.4)	5 (22.7)
Chemoradiotherapy, *n* (%)	9 (20.5)	4 (18.2)	5 (22.7)
Surgery + chemoradiotherapy, *n* (%)	6 (13.6)	4 (18.2)	2 (9.1)
Chemotherapy alone, *n* (%)	2 (4.6)	2 (9.1)	0 (0.0)
Radiotherapy alone, *n* (%)	1 (2.3)	0 (0.0)	1 (4.6)
**Treatment performed**			
Surgery, *n* (%)	32 (100.0)	16 (100.0)	16 (100.0)
Radiotherapy, *n* (%)	22 (75.9)	14 (87.5)	8 (61.5)
Systemic therapy, *n* (%)	13 (76.5)	9 (90.0)	4 (57.1)
-Immunotherapy, *n* (%)	4 (9.1)	4 (18.2)	0 (0.0)
**Adherence to recommended treatment, *n* (%)**	35 (79.5)	20 (90.9)	15 (68.2)
**Reason for deviation**			
Cancer progression, *n* (%)	5 (55.6)	1 (50.0)	4 (57.1)
Re-evaluation in Oncology department, *n* (%)	2 (22.2)	0 (00.0)	2 (28.6)
Patient refusal, *n* (%)	2 (22.2)	1 (50.0)	1 (14.3)

**Table 4:** Care waiting times for the entire study population and for each group.

	All Patients (*n =* 44) 1 March–31 August 2020	Group 1 (*n =* 22) 1 March–31 May 2020	Group 2 (*n =* 22) 1 June–31 August 2020
**All treatments**			
- **DMI ^1^**	***n* = 40**	***n* = 19**	***n* = 21**
Mean ± SD (days)	12.1 ± 7.7	12.3 ± 7.1	11.9 ± 8.1
Median [range] (days)	10 [1–34]	10 [1–28]	9 [1–34]
- **MTI ^2^**	***n* = 38**	***n* = 18**	***n* = 20**
Mean ± SD (days)	23.2 ± 16.3	19.7 ± 16.3	26.3 ± 15.8
Median [range] (days)	19 [4–68]	13.5 [5–62]	24 [4–68]
- **DTI ^3^**	***n* = 42**	***n* = 21**	***n* = 21**
Mean ± SD (days)	32.7 ± 18.1	29.0 ± 18.6	36.5 ± 16.7
Median [range] (days)	31 [5–79]	26 [5–79]	41 [12–75]
**Surgery first treatment**			
- **DMI**	***n* = 28**	***n* = 13**	***n* = 15**
Mean ± SD (days)	12.6 ± 8.0	12.9 ± 7.6	12.4 ± 8.4
Median [range] (days)	11.5 [1–34]	14 [1–28]	10 [1–34]
- **MTI**	***n* = 28**	***n* = 13**	***n* = 15**
Mean ± SD (days)	17.2 ± 13.0	12.3 ± 7.4	21.5 ± 15.1
Median [range] (days)	13 [4–68]	12 [5–29]	19 [4–68]
- **DTI**	***n* = 32**	***n* = 16**	***n* = 16**
Mean ± SD (days)	27.3 ± 15.6	21.9 ± 12.0	32.6 ± 17.0
Median [range] (days)	22.5 [5–75]	20.5 [5–44]	31.5 [12–75]
**Other first treatment**			
- **DMI**	***n* = 12**	***n* = 6**	***n* = 6**
Mean ± SD (days)	10.8 ± 6.6	10.8 ± 5.9	10.7 ± 7.2
Median [range] (days)	8 [1–23]	9 [3–20]	8 [1–23]
- **MTI**	***n* = 10**	***n* = 5**	***n* = 5**
Mean ± SD (days)	39.8 ± 12.9	39.0 ± 17.2	40.6 ± 6.0
Median [range] (days)	39 [19–62]	39 [19–62]	39 [34–49]
- **DTI**	***n* = 10**	***n* = 5**	***n* = 5**
Mean ± SD (days)	50.1 ± 14.2	51.4 ± 18.4	48.8 ± 7.6
Median [range] (days)	46.5 [26–79]	47 [26–79]	46 [42–63]

^1^ Diagnosis to multidisciplinary team meeting interval, ^2^ Multidisciplinary team meeting to treatment interval, ^3^ Diagnosis to treatment interval.

**Table 5:** Surgical management and reconstruction data for the entire study population and for each group.

	All Patients (*n =* 44) 1 March–31 August 2020	Group 1 (*n =* 22) 1 March–31 May 2020	Group 2 (*n =* 22) 1 June–31 August 2020
**Surgery decision, *n* (%)**	32 (72.7)	16 (72.7)	16 (72.7)
-Actually performed, n (%)	32 (100.0)	16 (100.0)	16 (100.0)
**Type of intervention**			
Glossectomy, *n* (%)	6 (18.8)	1 (6.3)	5 (31.3)
Pelvimandibulectomy, *n* (%)	6 (18.8)	4 (25.0)	2 (12.5)
Maxillectomy, *n* (%)	5 (15.6)	4 (25.0)	1 (6.3)
Pelviglosso mandibulectomy, *n* (%)	4 (12.5)	2 (12.5)	2 (12.5)
Parotidectomy, *n* (%)	3 (9.4)	2 (12.5)	1 (6.3)
Other, *n* (%)	8 (25.0)	3 (18.8)	5 (31.3)
-Oropharyngectomy, *n* (%)	1 (3.1)	0 (0.0)	1 (6.3)
-Excision of a cheek lesion, *n* (%)	2 (6.3)	0 (0.0)	2 (12.5)
-Nasal amputation, *n* (%)	2 (6.3)	2 (12.5)	0 (0.0)
-Labial amputation, *n* (%)	1 (3.1)	1 (6.3)	0 (0.0)
-Cervicotomy, *n* (%)	1 (3.1)	0 (0.0)	1 (6.3)
-Salvage lymph node dissection, *n* (%)	1 (3.1)	0 (0.0)	1 (6.3)
**Lymph node dissection, *n* (%)**	18 (56.3)	7 (43.8)	11 (68.8)
**Reconstruction, *n* (%)**	24 (75.0)	11 (68.8)	13 (81.3)
Immediate, *n* (%)	21 (87.5)	8 (72.7)	13 (100.0)
Secondary, *n* (%)	3 (12.5)	3 (27.3)	0 (0.0)
Double flap, *n* (%)	2 (8.3)	1 (9.1)	1 (7.7)
**Flap types**	***n* = 26**	***n* = 12**	***n* = 14**
Radial forearm, *n* (%)	10 (38.5)	3 (25.0)	7 (50.0)
Fibula, *n* (%)	6 (23.1)	3 (25.0)	3 (21.4)
Latissimus dorsi, *n* (%)	5 (19.2)	2 (16.7)	3 (21.4)
Scapula, *n* (%)	2 (7.7)	2 (16.7)	0 (0.0)
Other, *n* (%)	3 (11.5)	2 (16.7)	1 (7.1)
-Serratus, *n* (%)	1 (3.9)	1 (8.3)	0 (0.0)
-Pectoralis major (local), *n* (%)	1 (3.9)	0 (0.0)	1 (7.1)
-Mustarde (local), *n* (%)	1 (3.9)	1 (8.3)	0 (0.0)
**Flap success, *n* (%)**	23 (88.5)	10 (83.3)	13 (92.9)
**Hospitalization duration**			
Mean ± SD (days)	19.7 ± 17.5	19.9 ± 15.2	19.5 ± 19.6
Median [range] (days)	12 [2–79]	16 [3–51]	11 [2–79]
**Complications, *n* (%)**	9 (28.1)	4 (25.0)	5 (31.3)
Surgical re-intervention, *n* (%)	4 (12.5)	3 (18.8)	1 (6.3)
-Flap failure, *n* (%)	3 (9.4)	2 (12.5)	1 (6.3)
-Hemostasis verification, *n* (%)	1 (3.1)	1 (6.3)	0 (0.0)
Infection, *n* (%)	5 (15.6)	1 (6.3)	4 (25.0)
-Flap or surgical site, *n* (%)	2 (6.3)	1 (6.3)	1 (6.3)
-Pulmonary infection, *n* (%)	3 (9.4)	0 (0.0)	3 (18.8)
**Death in hospitalization, *n* (%)**	1 (3.1)	0 (0.0)	1 (6.3)

**Table 6:** SARS-CoV-2 testing and results for the entire study population and for each group.

	All Patients (*n =* 44) 1 March–31 August 2020	Group 1 (*n =* 22) 1 March–31 May 2020	Group 2 (*n =* 22) 1 June–31 August 2020
**Testing for SARS-CoV-2, *n* (%)**	20 (45.5)	6 (27.3)	14 (63.6)
**Method**			
PCR, *n* (%)	20 (100.0)	6 (100.0)	14 (100.0)
**Results**			
Positive, *n* (%)	0 (0.0)	0 (0.0)	0 (0.0)
Negative, *n* (%)	20 (100.0)	6 (100.0)	14 (100.0)

**Table 7:** Patients’ cancer status and survival at 6 and 12 months for the entire study population and for each group.

	All Patients (*n =* 44) 1 March–31 August 2020	Group 1 (*n =* 22) 1 March–31 May 2020	Group 2 (*n =* 22) 1 June–31 August 2020
**Cancer status (12 months)**			
Remission, *n* (%)	16 (38.1)	7 (33.3)	9 (42.9)
Progression/recurrence, *n* (%)	26 (61.9)	14 (66. 7)	12 (57.1)
**Patients survival**			
6 months, *n* (%)	29 (69.1)	16 (76.2)	13 (61.9)
12 months, *n* (%)	25 (59.5)	13 (61.9)	12 (57.1)

## Data Availability

The data presented in this study are available in the main text, figures and/or tables of the article. All data are also available on request from the corresponding author.
